# S100A9 Tetramers, Which are Ligands of CD85j, Increase the Ability of MVA_HIV_-Primed NK Cells to Control HIV Infection

**DOI:** 10.3389/fimmu.2015.00478

**Published:** 2015-09-23

**Authors:** Uriel Y. Moreno-Nieves, Céline Didier, Yves Lévy, Françoise Barré-Sinoussi, Daniel Scott-Algara, ANRS HIV Vaccine Network (AHVN)

**Affiliations:** ^1^Unité de Régulation des Infections Rétrovirales, Department of Virology, Institut Pasteur, Paris, France; ^2^INSERM U955, AP-HP, Groupe Henri-Mondor Albert-Chenevier, Immunologie Clinique, Creteil, France

**Keywords:** NK cell, dendritic cell, HIV-1, MVA-HIV, S100A9

## Abstract

Natural killer (NK) cells are the major antiviral effector population of the innate immune system. We previously found that S100A9 is a novel ligand of the receptor CD85j and that S100A9 tetramers enhance the anti-HIV activity of NK cells. Also, we found that dendritic cells (DCs) infected by the HIV vaccine candidate, MVA_HIV_, prime NK cells to specifically control HIV infection in autologous CD4^+^ T cells. In this study, we analyzed whether stimulation of NK cells by S100A9 tetramers prior to the priming by MVA_HIV_-infected DCs modulates the subsequent anti-HIV activity of NK cells. We found that S100A9 tetramers activate NK cells and that DCs enhance the anti-HIV activity of NK cells. Interestingly, we observed that stimulation of NK cells by S100A9 tetramers, prior to the priming, significantly increased the subsequent anti-HIV activity of NK cells and that the enhanced anti-HIV activity was observed following different conditions of priming, including the MVA_HIV_-priming. As S100A9 tetramers alone directly increase the anti-HIV activity of NK cells and as this increased anti-HIV activity is also observed following the interaction of NK cells with MVA_HIV_-infected DCs, we propose S100A9 tetramers as potential adjuvants to stimulate the anti-HIV activity of NK cells.

## Introduction

Natural killer (NK) cells are the major antiviral effector population of the innate immune system. They control viral infections and shape the adaptive immune responses (1–3). There is a bidirectional cross-talk between NK cells and dendritic cells (DCs), which results in DC-mediated NK cell activation and in NK cell-dependent DC maturation or apoptosis [reviewed in Ref. (4, 5)]. In homeostatic conditions, blood NK cells are in a resting state with low effector functions as evidenced by their lower responsiveness to target cells (6) or cytokines compared with activated NK cells. After activation by the cross-talk with DCs (7, 8) or by cytokines, NK cells become “primed,” which allow them to rapidly and strongly respond to a subsequent stimulation (6).

We previously reported that CD85j^+^ NK cells have significantly higher capacity to control HIV infection in autologous DCs compared with CD85j^−^ NK cells (9), and our studies suggested that the inhibition of HIV replication is due to the interaction of the receptor CD85j with a new ligand, the S100A9 molecule (10). In addition, we found that stimulation of NK cells by S100A9 tetramers significantly enhances the anti-HIV activity of NK cells (10). In other study, we found that the HIV vaccine candidate Modified Vaccinia Ankara encoding an HIV polypeptide (MVA_HIV_), developed by the French National Agency for Research on AIDS and viral hepatitis (ANRS), specifically primes NK cells to efficiently control HIV infection in autologous CD4^+^ T cells (11).

In this present study, we investigated whether stimulation of NK cells by S100A9 tetramers prior to the priming by MVA_HIV_-infected DCs modulates the ability of primed NK cell to control HIV infection in autologous CD4^+^ T cells.

## Materials and Methods

### Ethics statement

Blood was obtained from healthy donors through the French blood bank (Etablissement Français du Sang: EFS) in the setting of EFS-Institut Pasteur Convention. Donors signed informed consent forms allowing the use of their samples for clinical research according to French laws. Our study was approved externally (Etablissement Français du Sang) and internally (Biomedical Research Committee, Institut Pasteur).

### Isolation and differentiation of human primary cells

Peripheral blood mononuclear cells (PBMCs) were obtained from blood by Ficoll-Paque density gradient centrifugation according to manufacturer’s protocol. CD14^+^ monocytes were isolated from PBMC using anti-CD14 MicroBeads, LS+ columns and MACS separators (Miltenyi Biotec). Monocyte-derived DCs (referred as DCs) were differentiated from CD14^+^ monocytes by culture with rhIL-4 and rhGM-CSF (at 500 and 1,000 U/ml, respectively, R&D Systems) during 7 days. Sequentially after the CD14^+^ isolation, CD4^+^ T cells and NK cells were isolated using anti-CD4 and NK cells isolation MicroBeads, respectively, LS+ columns and MACS separators (Miltenyi Biotec). CD4^+^ T cells and NK cells were frozen in 90% fetal calf serum (FCS; PAA) and 10% DMSO (Sigma). The percentage of NK cells and CD4^+^ T cells after isolation was 95% by flow cytometry. We have previously determined that, in our settings, NK cell activity is not affected by freezing (12, 13). After thawing, NK cells were cultured overnight in medium prior to the priming. In all experiments, cells were cultured in DMEM supplemented with 10% FCS (PAA), 10 mM HEPES, 1× non-essential amino acids, 100 μg/ml streptomycin, 100 U/ml penicillin, 1 mM sodium pyruvate, 2 mM l-glutamine, 1× vitamins, and 50 μM 2-mercaptoethanol (Invitrogen).

### Viruses and infections

Modified Vaccinia Ankara encoding an HIV polypeptide (referred as MVA_HIV_) was developed by the ANRS, and it has been analyzed in preclinical studies (11, 14, 15). MVA_HIV_ encodes for a fusion polypeptide *gag-pol-nef* of HIV-1. MVA_WT_ is the wild-type vector. DCs were infected by either MVA_WT_ or MVA_HIV_ at a MOI of 0.25. CD4^+^ T cells were infected by R5 tropic HIV-1 *Bal* strain at a MOI of 10^−1^. Prior to HIV-1 infection, CD4^+^ T cells were stimulated for 4 days by PHA-L (1 μg/ml) and IL-2 (100 U/ml).

### Production of S100A9 proteins

S100A9 monomers were obtained from Tebu-bio (Paris, France). S100A9 tetramers were produced by Protenia (Dr El Yahyaoui, Ifrane, Morocco) using standard procedures. Briefly, S100A9 (Calgranuline B) was cloned in pET3a vector and, after verification of the insert, BL21(DE3) Origami *Escherichia coli* strain was transformed. Production of tetramer was tested after strain lysis, and protein purification was verified by SDS-PAGE gel. S100A9 tetramers were passed through endotoxin removal columns (Pierce). Proteins used in our experiments were LPS free.

### S100A9 stimulation and MVA-priming of NK cells

We established an *in vitro* coculture system, allowing the priming of NK cells by MVA-infected DCs (11, 15). As MVA is a non-replicating highly cytolytic virus, we infected or not DCs by MVA_WT_ or MVA_HIV_, and after 24 h (at the pic of expression of HIV antigens; Figure S1 in Supplementary Material) we added non-infected autologous DCs and NK cells at a final ratio of 5:5:1, respectively. The MVA-infected DC/DC/NK cell coculture was done for 4 days. In these conditions, the added non-infected DCs were able to phagocyte MVA-infected DCs and prime NK cells. To investigate the effect of S100A9 stimulation on the priming of NK cell by MVA-infected DCs, NK cells were incubated during 4 h with 1 μg/ml of S100A9 tetramers or S100A9 monomers and washed prior to the priming.

### Analysis of NK-cell activation

After a 4-h or a 4-day stimulation of NK cells by S100A9 proteins, or after a 4-h stimulation of NK cells by S100A9 proteins followed by 4 days of priming, NK cells were collected and the expression of CD69 was measured by flow cytometry on an LSRII instrument (BD Biosciences) on gated NK cells. The analysis was done using Kaluza™ v1.2 Software (Beckman Coulter) or FlowJo v10.0.8 (Tree Star).

### Analysis of intracellular cytokine production and CD107a expression by NK cells

Natural killer cells were stimulated by S100A9 proteins during 4 h and put in culture with DCs infected or not by MVA_HIV_ or MVA_WT_. Then, intracellular IFN-γ and surface CD107a expression (surrogate of degranulation) were assessed 4 h later, as previously described (16). Alternatively, after the 4-day priming, NK cells were harvested and cultured with HIV-infected CD4^+^ T cells at a ratio E/T of 1:5, and the expression of intracellular IFN-γ and TNF-α and surface CD107a on NK cells was determined. The acquisition was performed on an LSRII instrument (BD Biosciences). The analysis was done using Kaluza™ v1.2 Software (Beckman Coulter).

### DC maturation during the NK/DC coculture

Dendritic cells were infected or not by MVA_HIV_ or MVA_WT_, and 24 h later non-infected DCs and NK cells were added at a final ratio of 5:5:1, respectively. The MVA-infected DC/DC/NK cell coculture was done for 4, 24, 48, or 96 h. Then, the supernatant was frozen, and cells were resuspended in PBS. DC maturation was determined by the expression of CD83 and CD80 by flow cytometry using an LSRII instrument (BD Biosciences). The analysis was done using Kaluza™ v1.2 Software (Beckman Coulter) or FlowJo v10.0.8 (Tree Star).

### Analysis of the anti-HIV activity of primed NK cell

After 4 days of priming, NK cells were harvested and cultured with HIV-infected autologous CD4^+^ T cells. The ability of primed NK cells to control HIV infection was determined at day 10. To this end, we analyzed the percentage of HIV-infected CD4^+^ T cells in culture with primed NK cells, by measuring the expression of intracellular HIV-1 p24 Ag in gated CD4^+^ T cells by flow cytometry using a Gallios instrument (Beckman Coulter). The analysis was done using Kaluza™ v1.2 Software (Beckman Coulter) or FlowJo v10.0.8 (Tree Star).

### Statistical analysis

The results are expressed as mean ± SE. For statistical analysis, Wilcoxon matched-pairs *t*-test was performed. *p* values are shown when statistical significance was reached.

## Results

### Stimulation of NK cells by S100A9 tetramers activates NK cells

**Figure 1:**
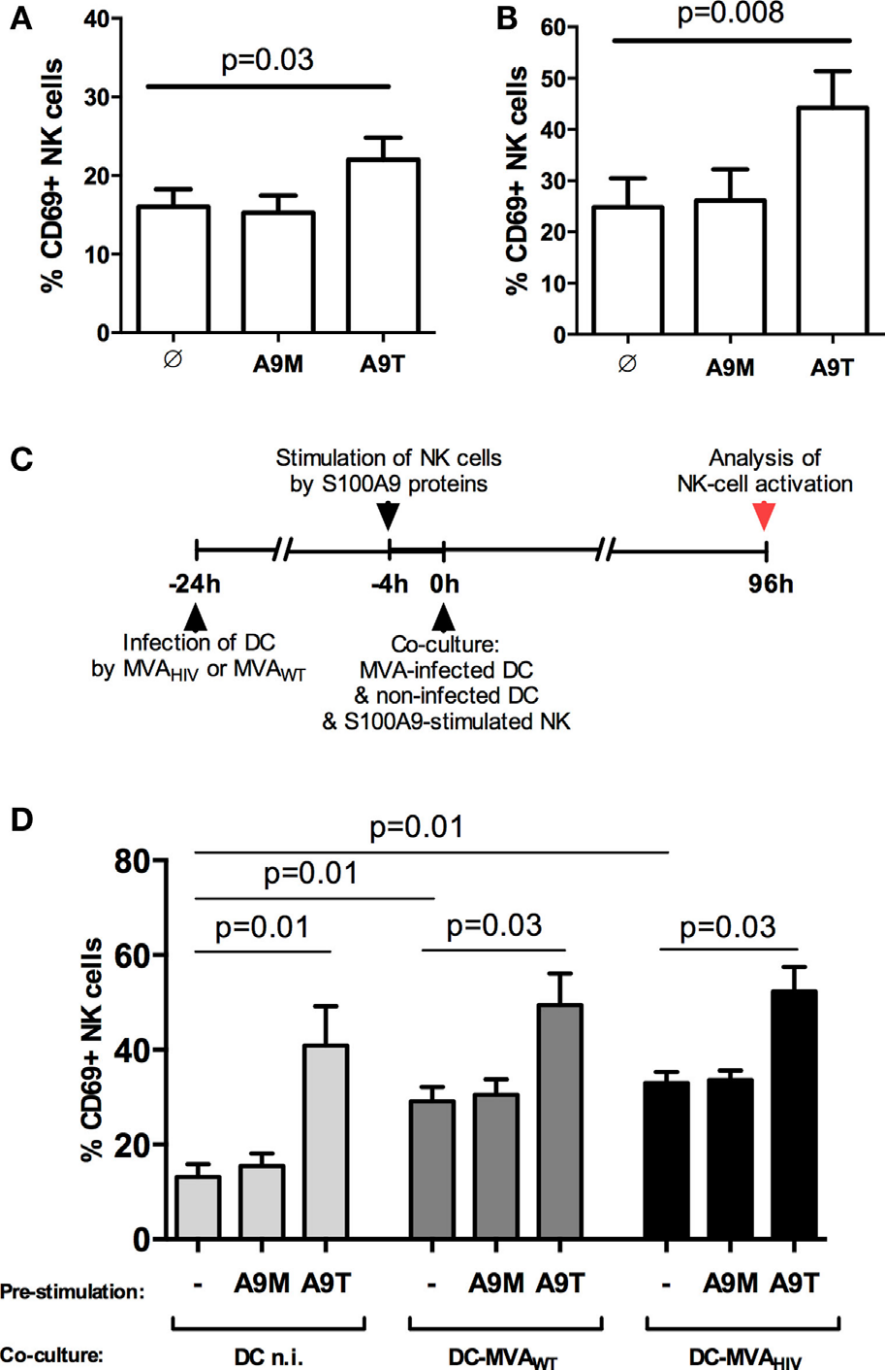
**S100A9 tetramers enhance NK-cell activation**. **(A)** Purified NK cells were stimulated by S100A9 tetramers or S100A9 monomers at 1 μg/ml during 4 h, then CD69 expression was assessed; graph shows cumulative results from five independent experiments. **(B)** Purified NK cells were stimulated by S100A9 tetramers or S100A9 monomers at 1 μg/ml during 4 days, then CD69 expression was assessed; graph shows cumulative results from seven independent experiments. **(C)** Schema depicts the protocol used in **(D)**, in brief: DCs were infected or not by MVA_WT_ or MVA_HIV_, and 24 h later non-infected DCs and S100A9-stimulated NK cells were added to the culture; finally, 96 h (4 days) later, CD69 expression on NK cells was analyzed. **(D)** Graph shows cumulative results from five independent experiments. Results are expressed as mean ± SE and *p* values are shown. A9M, S100A9 monomer; A9T, S100A9 tetramer; DCn.i., non-infected DC; DC-MVA_WT_, MVA_WT_-infected DC; DC-MVA_HIV_, MVA_HIV_-infected DC.

We previously showed that stimulation of NK cells by S100A9 tetramers enhances the early responsiveness against target cells and the control of HIV infection in CD4^+^ T cells (10). Here, we first analyzed whether S100A9 tetramers modulate the activation of NK cells (Figure 1). We observed that 4-h stimulation of PBMCs (data not shown) or purified NK cells (Figure 1A) by S100A9 tetramers enhanced the activation of NK cells, assessed by the expression of the activation marker CD69, whereas stimulation of NK cells by S100A9 monomers had no effect. Moreover, we observed that 4-day stimulation of NK cells by S100A9 tetramers also resulted in higher NK-cell activation (Figure 1B).

Previously, we established an *in vitro* coculture system allowing the priming of NK cells by autologous DCs infected by MVA_HIV_ (11). In these settings, we observed that MVA_HIV_-primed NK cells have significantly higher capacity to control HIV infection in CD4^+^ T cells compared with NK cells primed by non-infected DCs or MVA_WT_-infected DCs (11). Here, using this coculture system, we analyzed whether the stimulation of NK cells by S100A9 proteins prior to the priming impacts the level of NK-cell activation at the end of the priming (Figure S2 in Supplementary Material and Figures 1C,D). Interestingly, we found that stimulation of NK cells by S100A9 tetramers prior to the priming resulted in higher NK-cell activation after the 4-day priming (Figure 1D), and the level of CD69 expression on S100A9-tetramer-stimulated NK cells was similar independently of the priming conditions (non-infected, MVA_WT_- or MVA_HIV_-infected DCs; Figure 1D). Pre-stimulation of NK cells by S100A9 monomers did not modify the subsequent NK-cell activation. In summary, stimulation of NK cells by S100A9 tetramers, but not S100A9 monomers, increased the activation of NK cells, and the increased NK-cell activation was maintained during the priming of NK cells by either non-infected or MVA-infected DCs.

### Pre-stimulation of NK cells by S100A9 tetramers moderately changes NK-cell responses during the priming

**Figure 2:**
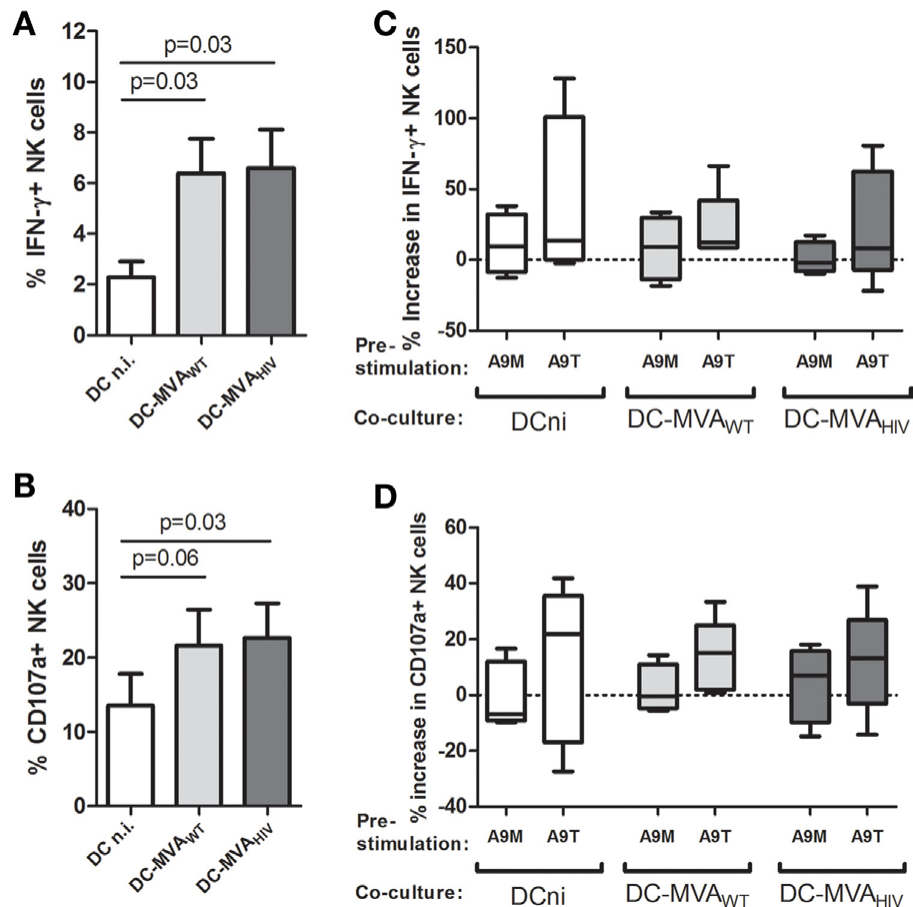
**Following S100A9-tetramer stimulation, NK cells moderately respond higher against DCs**. NK cells were stimulated or not by S100A9 tetramers or S100A9 monomers at 1 μg/ml during 4 h, then NK cells were cultured with DCs infected or not by MVA_WT_ or MVA_HIV_. After 4 h of coculture, the degranulation (CD107a surface expression) and intracellular IFN-γ production were analyzed on gated NK cells. **(A,B)** Graphs show the percentage of IFN-γ^+^**(A)** and CD107a^+^**(B)** NK cells; cumulative results from four independent experiments are shown. Results are expressed as mean ± SE and *p* values are shown. **(C,D)** Graphs show the percentage increase in IFN-γ^+^**(C)** and CD107a^+^**(D)** expression on S100A9-stimulated NK cells compared with unstimulated NK cells; cumulative results from four independent experiments are shown. Results are expressed as whiskers with minimum and maximum. A9M, S100A9 monomer; A9T, S100A9 tetramer; DCni, non-infected DC; DC-MVA_WT_, MVA_WT_-infected DC; DC-MVA_HIV_, MVA_HIV_-infected DC.

Next, we investigated whether S100A9 tetramers modulate NK-cell and DC responses during the priming. We analyzed the early cytokine production and degranulation of S100A9-stimulated NK cells against DCs infected or not by MVA (Figure 2). As previously described (15), we observed that NK cells produce more intracellular IFN-γ (Figure 2A) and higher degranulation (Figure 2B) against MVA_WT_- and MVA_HIV_-infected DCs compared with non-infected DCs and that stimulation of NK cells by S100A9 tetramers trended to enhance IFN-γ production (Figure 2C) and degranulation (Figure 2D). Pre-stimulation of NK cells by S100A9 monomers had no effect on the early NK-cell response to DCs. Study of the NK-cell receptor repertoire expression at different time points during the coculture, from 12 to 96 h (Figure S3 in Supplementary Material and data not shown), showed that stimulation of NK cells by S100A9 tetramers, but not S100A9 monomers, resulted in a trended lower expression of NKG2C (Figure S3A in Supplementary Material) and trended decreased expression of NKG2D (Figure S3B in Supplementary Material). The analysis of NK-cell proliferation showed that pre-stimulation of NK cells by S100A9 monomers or S100A9 tetramers did not modify NK-cell proliferation (data not shown).

As S100A9-tetramer-stimulated NK cells trended to produce more IFN-γ (Figure 2C), and as IFN-γ is implicated in the NK-cell-mediated DC maturation (17, 18), we analyzed the maturation of DCs at different time points during the priming, from 4 to 96 h (Figure S4 in Supplementary Material). DC maturation was evaluated by the expression of maturation marker CD83 (Figures S4A,C in Supplementary Material) and costimulatory molecules CD80 (Figures S4B,D in Supplementary Material) and CD86 (data not shown) on DCs. We found that during the coculture, the maturation of MVA_WT_- and MVA_HIV_-infected DCs was higher compared with the maturation of non-infected DCs; however, we did not observe a significant modification of DC maturation when DCs were cultured with S100A9-stimulated NK cells. Finally, as we observed that S100A9 tetramers activate NK cells (Figure 1), and as activated NK cells can lyse DCs (19, 20), we measured DC death during the coculture; however, we did not find any difference in the level of dead DCs in culture with unstimulated or S100A9-stimulated NK cells (Figure S5 in Supplementary Material).

Overall, we found that besides the significant increase in NK-cell activation (Figure 1), stimulation of NK cells by S100A9 tetramers resulted only in a modest modulation of IFN-γ production, degranulation, and repertoire expression on NK cells and had no impact on DC maturation and NK-cell proliferation.

### S100A9-tetramer stimulation prior to the priming enhances the subsequent anti-HIV response of primed NK cells

**Figure 3:**
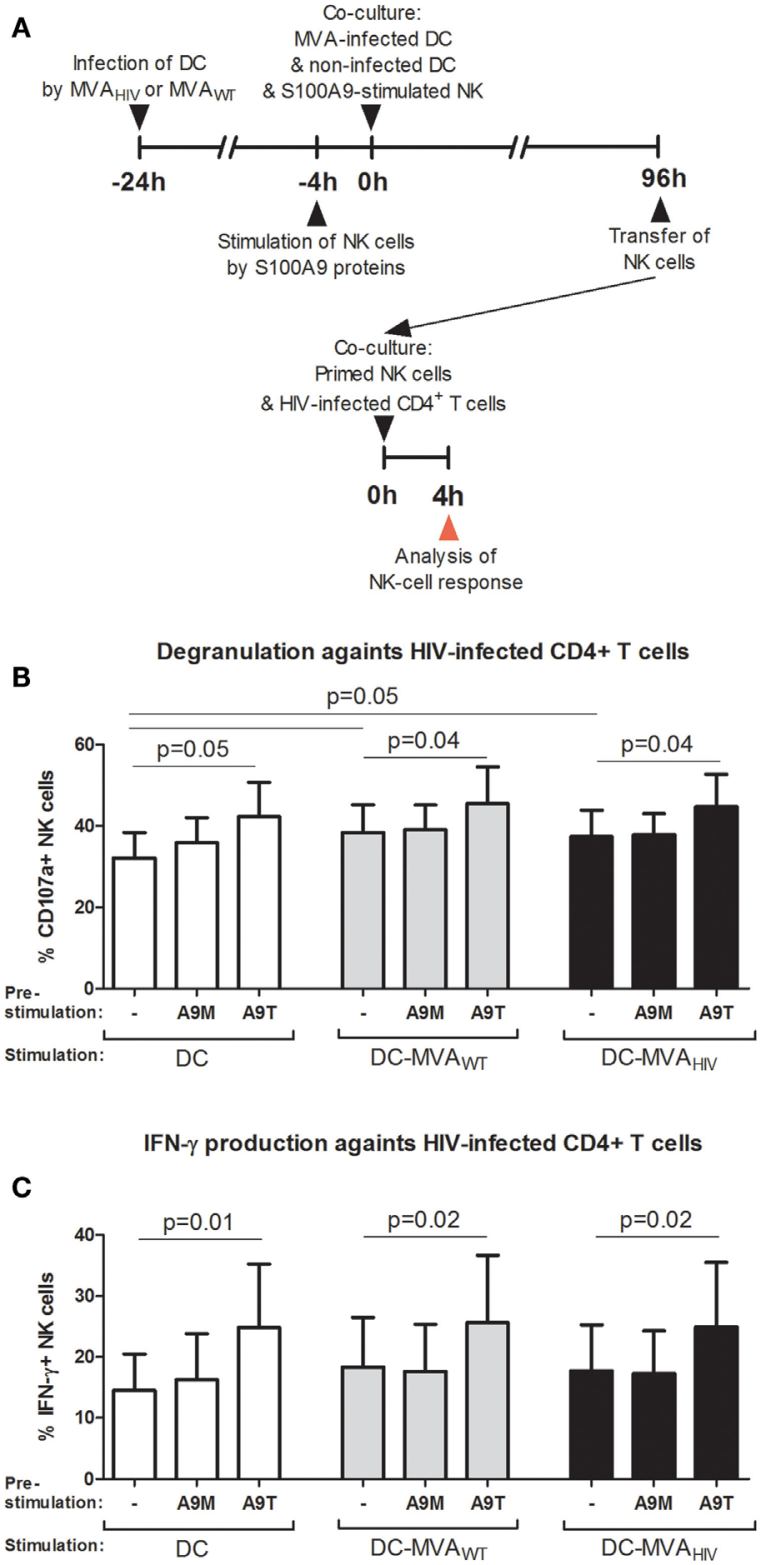
**S100A9-tetramer stimulation prior to the priming by DCs enhances the anti-HIV response of NK cells**. S100A9-stimulated and MVA-primed NK cells were tested in their ability to degranulate (express CD107a) and produce IFN-γ in response to HIV-infected autologous CD4^+^ T cells. **(A)** Schema depicts the protocol used in **(B,C)**, in brief: DCs were infected or not by MVA_WT_ or MVA_HIV_, and 24 h later non-infected DCs and S100A9-stimulated NK cells were added to the culture; and 96 h later (4 days) NK cells were transferred to a culture of HIV-infected CD4^+^ T cells and the degranulation and IFN-γ production were assessed. **(B,C)** Graphs show the percentage of CD107a^+^**(B)** and IFN-γ^+^**(C)** NK cells; cumulative results from six independent experiments are shown as mean ± SE and *p* values are shown. A9M, S100A9 monomer; A9T, S100A9 tetramer; DC-MVA_WT_, MVA_WT_-infected DC; DC-MVA_HIV_, MVA_HIV_-infected DC.

Natural killer cells use several mechanisms to control viral infections, such as the secretion of antiviral cytokines (e.g., IFN-γ) and the release of lytic proteins contained in granules (i.e., degranulation). Therefore, we analyzed the capacity of NK cells primed under the above-mentioned conditions to degranulate and produce cytokines against HIV-infected autologous CD4^+^ T cells (Figure 3). Briefly, after the 4-day priming, NK cells were collected and incubated with HIV-infected CD4^+^ T cells (Figure 3A), and the degranulation (CD107a expression; Figure 3B) and the production of IFN-γ (Figure 3C) and TNF-α (data not shown) were assessed. As we previously reported (11), NK cells primed by MVA_WT_- and MVA_HIV_-infected DCs degranulate higher (Figure 3B) and trended to produce more IFN-γ (Figure 3C) against HIV-infected CD4^+^ T cells compared with NK cells primed by non-infected DCs. Interestingly, we observed that pre-stimulation of NK cells by S100A9 tetramers, but not S100A9 monomers, resulted in significantly increased degranulation (Figure 3B) and IFN-γ production (Figure 3C) after the priming, independently of the priming condition. Pre-stimulation of NK cells by S100A9 proteins did not modulate the TNF-α production by NK cells in response to HIV-infected CD4^+^ T cells (data not shown). Additionally, in these conditions, we analyzed the NK-cell response against the MHC-I-deficient K562 target cell (data not shown); we found that pre-stimulation of NK cells by S100A9 monomers or S100A9 tetramers did not modulate the NK-cell response against K562 cells in terms of degranulation and cytokine production. Overall, these results showed that stimulation of NK cells by S100A9 tetramers prior to the priming by DCs lead to increased early NK-cell response against HIV-infected CD4^+^ T cells after the priming.

### The enhanced control of HIV infection, induced by the stimulation of NK cells by S100A9 tetramers, is observed after the priming

**Figure 4:**
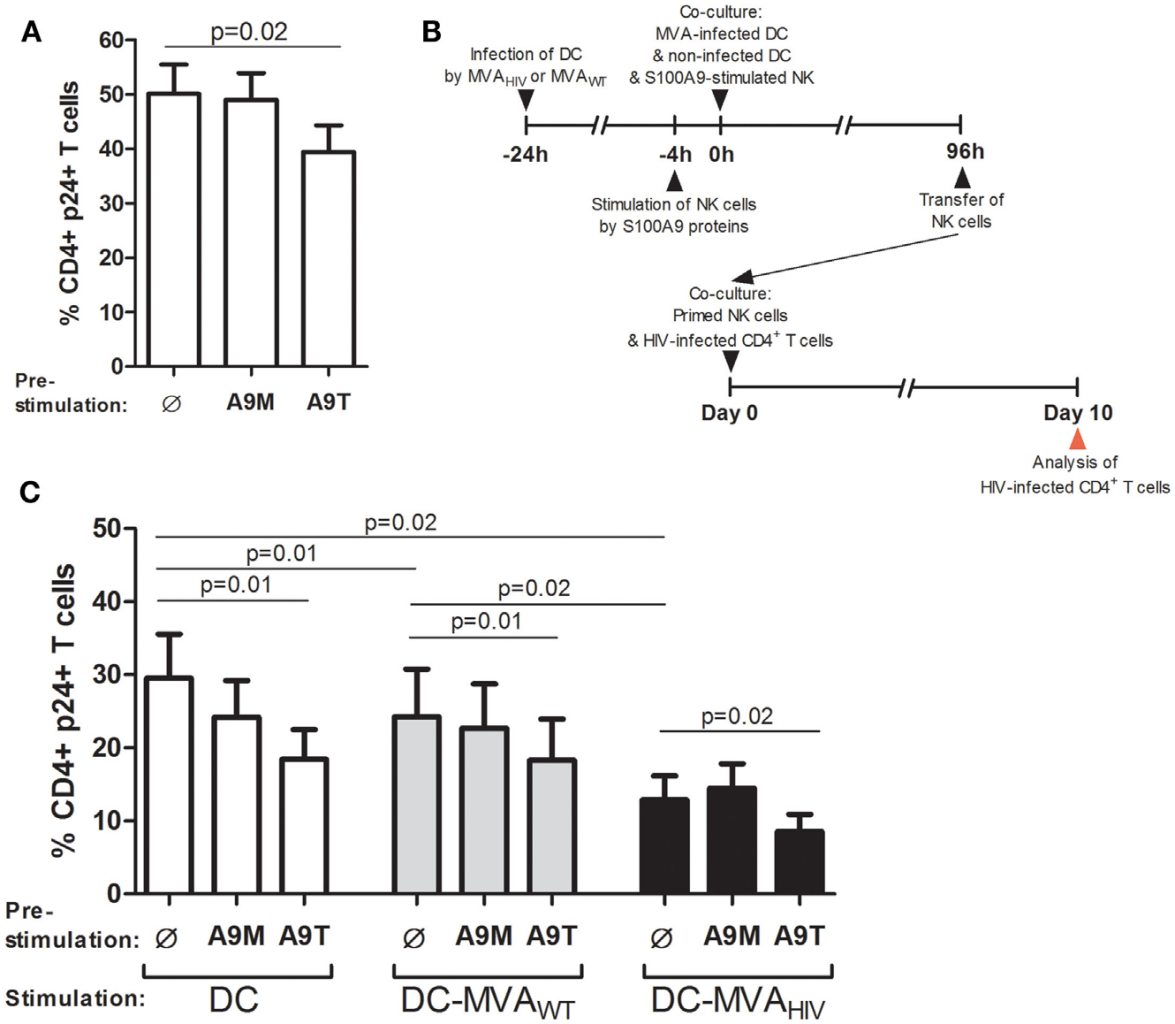
**S100A9-tetramer stimulation and MVA_HIV_-priming significantly enhance by ability of NK cells to control HIV infection**. S100A9-stimulated NK cells were tested in their ability to control HIV infection in autologous CD4^+^ T cells. **(A)** NK cells were stimulated or not by S100A9 tetramers or monomers at 1 μg/ml during 4 h and cultured with HIV-infected CD4^+^ T cells, and after 10 days of culture, the percentage of intracellular HIV-1 p24^+^ CD4^+^ T cells was analyzed by flow cytometry; graph shows cumulative results from six independent experiments. **(B)** Schema depicts the protocol used in **(C)**, in brief: DCs were infected or not by MVA_WT_ or MVA_HIV_, and 24 h later non-infected DCs and S100A9-stimulated NK cells were added to the culture; and 96 h later (4 days) NK cells were transferred to a culture of HIV-infected CD4^+^ T cells and the ability of NK cells to control HIV infection was assessed at 10 days post-HIV infection. **(C)** Graph shows the percentage of HIV p24^+^ CD4^+^ T cells in culture; cumulative results from eight independent experiments are expressed as mean ± SE and *p* values are shown. A9M, S100A9 monomer; A9T, S100A9 tetramer; DC-MVA_WT_, MVA_WT_-infected DC; DC-MVA_HIV_, MVA_HIV_-infected DC.

Then, we sought to determine the capacity of NK cells to control HIV infection in CD4^+^ T cells after stimulation by S100A9 tetramers and priming by DCs (Figure 4). We assessed the ability of NK cells to control HIV infection in CD4^+^ T cells by analyzing the percentage of HIV-1 p24-positive (HIV p24^+^) CD4^+^ T cells in culture with primed NK cells, at day 10 post HIV infection. As previously described (10), we found that stimulation of NK cells by S100A9 tetramers, but not S100A9 monomers, enhanced the capacity of NK cells to control HIV infection (Figure 4A).

In order to test the ability of NK cells stimulated by S100A9 proteins and primed by DCs to control HIV infection in CD4^+^ T cells, NK cells were pre-stimulated by S100A9 proteins and cultured during 4 days with DCs infected or not by MVA_WT_ or MVA_HIV_, then NK cells were collected and incubated with HIV-infected CD4^+^ T cells, and after 10 days the percentage of HIV p24^+^ CD4^+^ T cells in the culture was measured (Figure 4B). Of interest, we found that culture of NK cells with non-infected DCs during 4 days resulted in reduced percentage of HIV p24^+^ CD4^+^ T cells compared with unstimulated NK cells (Figure S6 in Supplementary Material), indicating that DCs alone naturally enhanced the ability of NK cells to control HIV infection in CD4^+^ T cells. According to our previous study (11), we observed that MVA_WT_- and MVA_HIV_-primed NK cells significantly decreased the percentage of HIV p24^+^ CD4^+^ T cells in culture compared with NK cells primed by non-infected DCs (Figure 4C), and among these priming conditions, MVA_HIV_-primed NK cells had the highest capacity to control HIV infection. Of note, we observed that in the absence of NK cells, culture of DCs (infected or not by MVA) with HIV-infected CD4^+^ T cells did not modify the percentage of HIV-infected CD4^+^ T cells (Figure S7 in Supplementary Material), further suggesting a role for NK cells in the control of HIV infection.

Interestingly, we observed that pre-stimulation of NK cells by S100A9 tetramers, but not S100A9 monomers, resulted in significantly lower percentage of HIV p24^+^ CD4^+^ T cells in all priming conditions (Figure 4C), indicating higher ability of S100A9-tetramer-stimulated NK cells to control HIV infection. Moreover, stimulation of DCs by S100A9 tetramers did not modify DC maturation (data not shown) and did not lower HIV infection in cocultured CD4^+^ T cells (Figure S8 in Supplementary Material). In addition, we analyzed the viability of CD4^+^ T cells in culture with primed NK cells (Figure S9 in Supplementary Material). We observed that priming of NK cells by non-infected DCs resulted in higher death of CD4^+^ T cells compared with priming of NK cells by MVA-infected DCs, likely due to the higher level of HIV infection. We also observed that most HIV p24^+^ CD4^+^ T cells were viable (Figure S9A in Supplementary Material). In summary, these results showed that stimulation of NK cells by S100A9 tetramers enhances the ability of NK cells to control HIV infection and also that this enhanced anti-HIV activity is observed in the context of NK/DC interactions, including the specific priming of NK cells by MVA_HIV_-infected DCs.

## Discussion

In this study, we showed that S100A9 tetramers, which have been demonstrated to be ligands of the receptor CD85j and enhance the anti-HIV activity of NK cells (10), are able to activate NK cells and increase their anti-HIV activity after the MVA priming. Using an *in vitro* system to prime NK cell by DCs, we observed that stimulation of NK cells by S100A9 tetramers results in enhanced NK-cell activation and enhanced anti-HIV activity after priming of NK cells. And interestingly, we found that stimulation of NK cells by S100A9 tetramers enhances the ability of MVA_HIV_-primed NK cells to control HIV infection.

In line with our previous report showing that stimulation of NK cells by S100A9 tetramers enhances the responsiveness of NK cells against target cells and increases their ability to control HIV infection in autologous CD4^+^ T cells (10), here we observed that S100A9 tetramers significantly activate NK cells. However, although S100A9-tetramer-stimulated NK cells display enhanced activation, they only trend to higher respond against DCs (infected or not by MVA) in terms of cytokine production and degranulation, suggesting that S100A9 tetramers do not significantly enhance the broad NK-cell responsiveness.

Regarding the NK/DC interactions, we found that culture of NK cells with autologous DCs significantly enhances the ability of NK cells to control HIV infection in CD4^+^ T cells. It has been well described that DCs enhance NK-cell functions as part of the NK/DC cross-talk (7, 17, 19, 21); however, to our knowledge this is the first report showing that DCs naturally enhance the anti-HIV activity of autologous NK cells. In our culture conditions, we found that stimulation of NK cells by S100A9 tetramers prior to the priming by DCs enhanced the NK-cell activation and the ability of NK cells to control HIV infection. And interestingly, regarding our previous demonstration that MVA_HIV_-infected DCs specifically prime NK cells to efficiently control HIV infection (11), here we found that S100A9 tetramers further enhance the ability of MVA_HIV_-primed NK cells to control HIV infection.

We previously reported that stimulation of NK cells by S100A9 tetramers enhances the NK-cell response against K562 target cells (10). However, here we did not observe enhanced NK-cell response against K562 target cells after the priming of S100A9-tetramer-stimulated NK cells, which is in contrast with the enhanced anti-HIV activity of NK cells pre-stimulated by S100A9 tetramers. One possible explanation for this observation could be that S100A9-tetramer-stimulated NK cells may up-regulate activating receptors (different from those analyzed) or death-receptor ligands (such as FasL or TRAIL) allowing them to respond against HIV-infected cells (22) but not against K562 cells (23, 24). Further experimental work is needed to delineate this issue.

Among the phenotypic changes of S100A9-tetramer-stimulated NK cells after the priming, in addition to higher activation, we observed trended lower NKG2C expression. Although up-regulation of NKG2C expression on NK cells has been reported during HIV infection (25), mainly driven by CMV coinfection (26), lower expression of NKG2C has been also associated with higher percentages of CD4^+^ T cells in HIV-infected children (27) and lower disease progression (28). It is therefore possible that in our conditions, NK-cell activation and lower NKG2C expression could be linked to the enhanced anti-HIV activity of NK cells. However, as all S100A9-tetramer-stimulated NK cells had a similar level of activation and NKG2C expression at the end of the priming, and as NK cells stimulated by S100A9 tetramers and primed by MVA_HIV_-infected DCs displayed the highest ability to control HIV infection in CD4^+^ T cells, we speculate that additional mechanisms other than NK-cell activation and NKG2C-triggering might be implicated.

A question raised by our data is how the enhanced NK-cell activation and anti-HIV activity, induced by S100A9-tetramer stimulation, are maintained after the 4-day priming of NK cells (and after different conditions of priming). One possible explanation is that the stimulation of NK cells by S100A9 tetramers modulates DC polarization or DC maturation (independently of the expression of maturation marker and costimulatory molecules), which in turn differently or further stimulates NK cells. In this line, it is worth to be noted that DCs with similar expression of costimulatory molecules can have different abilities to stimulate T cells (29) and that NK-cell function can be modified by different stimulatory signals (30, 31). Alternatively, one hypothesis to explain the enhanced NK-cell activation and anti-HIV activity of S100A9-tetramer-stimulated NK cells, after the priming by DCs, is that the NK/DC cross-talk acts as a “rheostat” by maintaining the differences in the level of NK-cell activation and effector function. This hypothesis is interesting considering that MVA-infected DCs express significantly lower S100A9 (32), therefore highlighting the effect of the pre-stimulation of NK cells by S100A9 tetramers. Although further investigation is required to assess this issue, our data highlight that short stimulations of NK cells before NK/DC interactions importantly impact the NK-cell activity several days later.

In mice, it has been shown that subsets of NK cells display memory-like features following antigen stimulation (33, 34). NK-cell memory has not yet been established in humans; however, recently it was observed that macaque NK cells display memory-like responses following SIV infection or vaccination (35). This observation in primates is of interest and opens the question of whether NK-cell stimulation could be relevant in human HIV vaccination. Although memory NK cells require investigation as well as their potential use in prophylactic vaccination, it is conceivable that stimulation of NK cells by vaccines and/or adjuvants in the settings of therapeutic vaccination (in individuals who are already infected by HIV) could directly increase the amount of NK cells responding to HIV as well as their anti-HIV potential.

We found that S100A9 tetramers alone enhance the anti-HIV activity of NK cells (10), and here we showed that they can also enhance the anti-HIV activity of NK cells following NK/DC interactions in the presence of an HIV vaccine candidate (MVA_HIV_). The mechanism by which NK cells control HIV replication in target cells is still unknown. We previously showed that control of HIV replication in DC by NK cells is mediated by a non-cytolytic mechanism. Along the same lines, evidence presented here suggested that NK cells are not killing HIV-infected CD4 T cells. Thus, altogether, these results suggest that NK cell can regulate HIV target cells in order to increase abortive replication. Therefore, from these observations we can hypothesize that S100A9 tetramers could directly stimulate NK cells to respond against HIV or adjuvant the anti-HIV activity of NK cells in the settings of MVA-based vaccinations. In addition, as NK cells play an important role at the interphase between the innate and adaptive immune system (36, 37), it is possible that stimulation of NK cells by S100A9 tetramers may have an impact on a broader system; hence, it would be interesting to analyze the impact of this NK-cell stimulation on the T-cell responses, which play an important role in protection following vaccination.

## Conclusion

Our observation that the stimulation of NK cells by S100A9 tetramers significantly increases the control of HIV, and that the higher control of HIV is also observed after the priming of NK cells by the HIV vaccine candidate MVA_HIV_, is of high interest. The use of adjuvants to stimulate NK-cell responses during vaccination has been poorly studied. According to our data, S100A9 tetramers could act as adjuvants to enhance the anti-HIV activity of NK cells.

## Author Contributions

UM-N designed and performed research, analyzed data, and wrote the article. CD performed research. YL designed research. FB-S designed research and analyzed data. DS-A designed research, analyzed data, and wrote the article. All authors read and approved the final article.

## Conflict of Interest Statement

The authors declare that the research was conducted in the absence of any commercial or financial relationships that could be construed as a potential conflict of interest.

## Supplementary Material

The Supplementary Material for this article can be found online at http://journal.frontiersin.org/article/10.3389/fimmu.2015.00478
