# Supplementary material for: S100A9 Tetramers, Which are Ligands of CD85j, Increase the Ability of MVAHIV-Primed NK Cells to Control HIV Infection
Source: Front Immunol. 2015 Sep 23;6:478. doi: 10.3389/fimmu.2015.00478 (PMC4585218; doi:10.3389/fimmu.2015.00478)
Supplement: Supplementary file 1 [file Image_1.PDF]

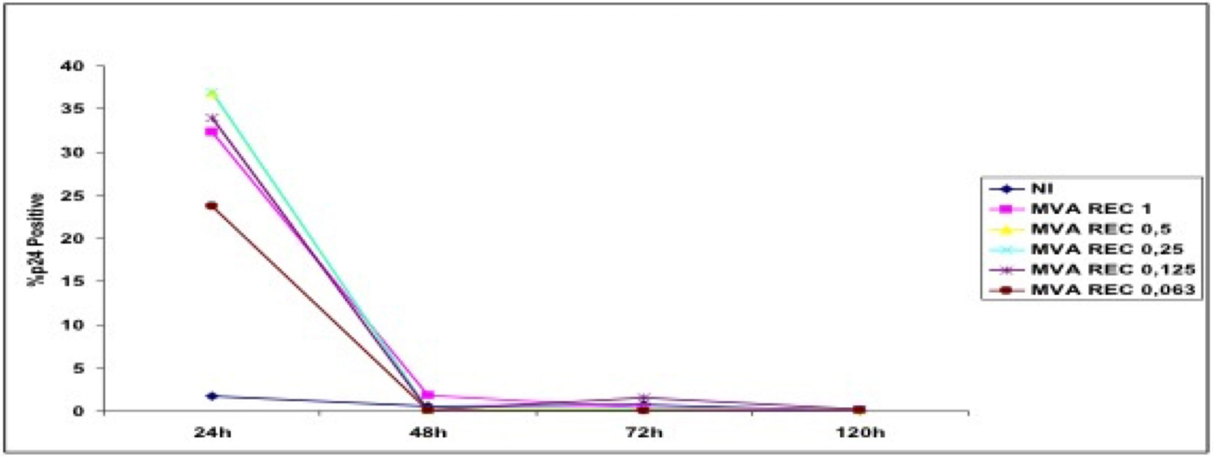

Figure S1 | HIV p24 expression on MVAHIV-infected DCs.
Monocytes were differentiated into DCs by 7 days of culture with IL-4 and GM-CSF. DCs were infected by MVAHIV at different MOIs: 0.063, 0.125, 0.25, 0.5 and 1. Then, intracellular expression of HIV p24 was measured at 24, 48, 72 and 120 hours post-infection. Graph shows a representative experiment.
