# Supplementary material for: S100A9 Tetramers, Which are Ligands of CD85j, Increase the Ability of MVAHIV-Primed NK Cells to Control HIV Infection
Source: Front Immunol. 2015 Sep 23;6:478. doi: 10.3389/fimmu.2015.00478 (PMC4585218; doi:10.3389/fimmu.2015.00478)
Supplement: Supplementary file 2 [file Image_2.PDF]

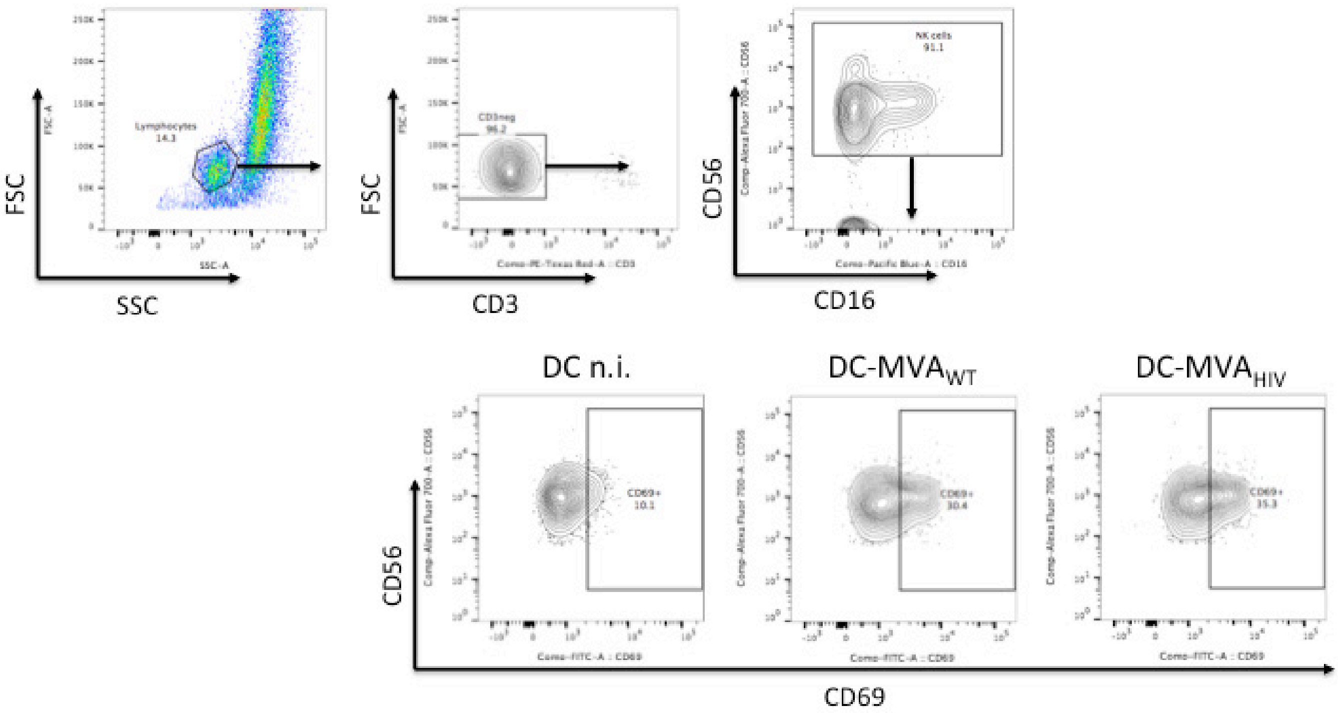

Figure S2 | NK-cell gating strategy and CD69 expression.
NK cells were cultured with DCs infected or not by MVAWT or MVAHIV, then, CD69 expression was analyzed on gated NK cells. NK cells were defined as CD3-CD56+CD16+/-. A representative example is shown. DC n.i.: non-infected DC; DC-MVAWT: MVAWT-infected DC; DC-MVAHIV: MVA-HIV-infected DC.
