# Supplementary material for: S100A9 Tetramers, Which are Ligands of CD85j, Increase the Ability of MVAHIV-Primed NK Cells to Control HIV Infection
Source: Front Immunol. 2015 Sep 23;6:478. doi: 10.3389/fimmu.2015.00478 (PMC4585218; doi:10.3389/fimmu.2015.00478)
Supplement: Supplementary file 3 [file Image_3.PDF]

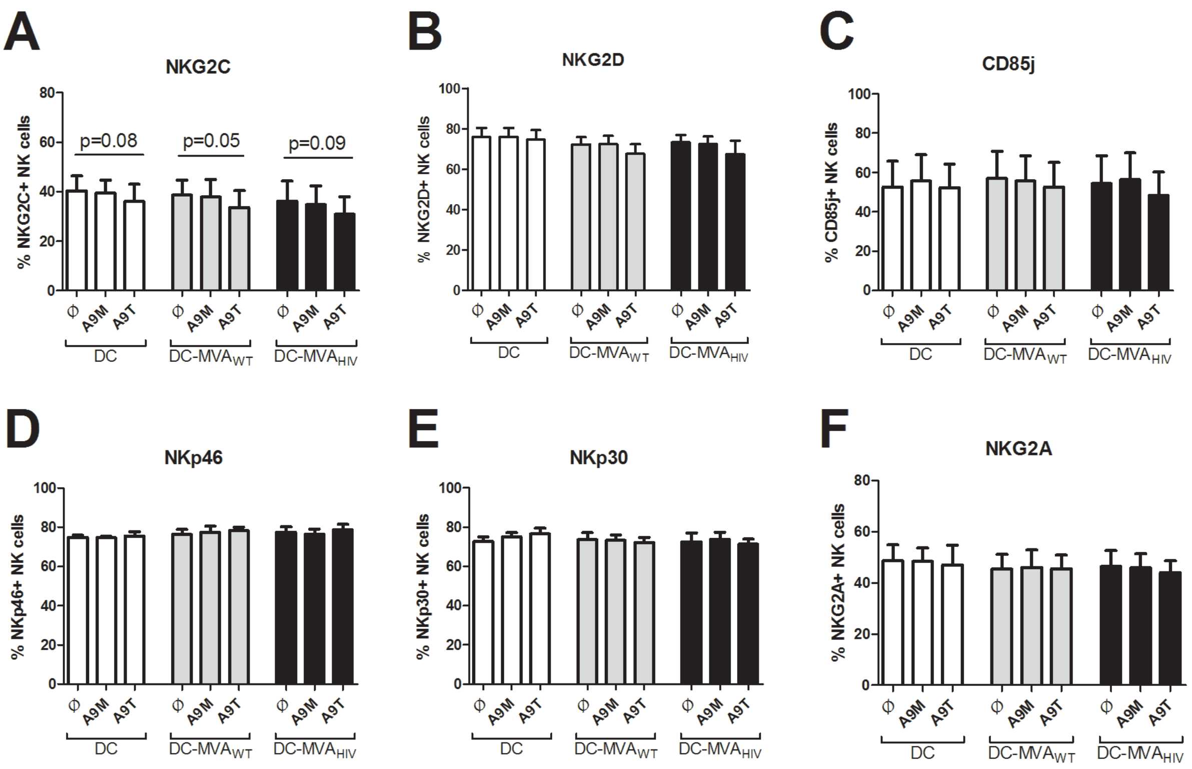

Figure S3 | The NK-cell receptor repertoire is moderately modified by S100A9-tetramer pre-stimulation.

NK cells were stimulated or not by S100A9 tetramers or SA00A9 monomers at 1μg/mL during 4 hours, and cultured with DCs infected or not by MVAWT or MVAHIV, during 4 days. Then the expression of the NK-cell receptor repertoire was analyzed by flow cytometry on gated NK cells. The expression of NKG2C (A), NKG2D (B), CD85j (C), NKp46 (D), NKp30 (E) and NKG2A (F) are shown; graphs represent cumulative results from at least 5 independent experiments expressed as mean ± SE. p values are shown. A9M: S100A9 monomer; A9T: S100A9 tetramer; DC-MVAWT: MVAWT-infected DC; DC-MVAHIV: MVAHIV-infected DC.
