# Supplementary material for: S100A9 Tetramers, Which are Ligands of CD85j, Increase the Ability of MVAHIV-Primed NK Cells to Control HIV Infection
Source: Front Immunol. 2015 Sep 23;6:478. doi: 10.3389/fimmu.2015.00478 (PMC4585218; doi:10.3389/fimmu.2015.00478)
Supplement: Supplementary file 4 [file Image_4.PDF]

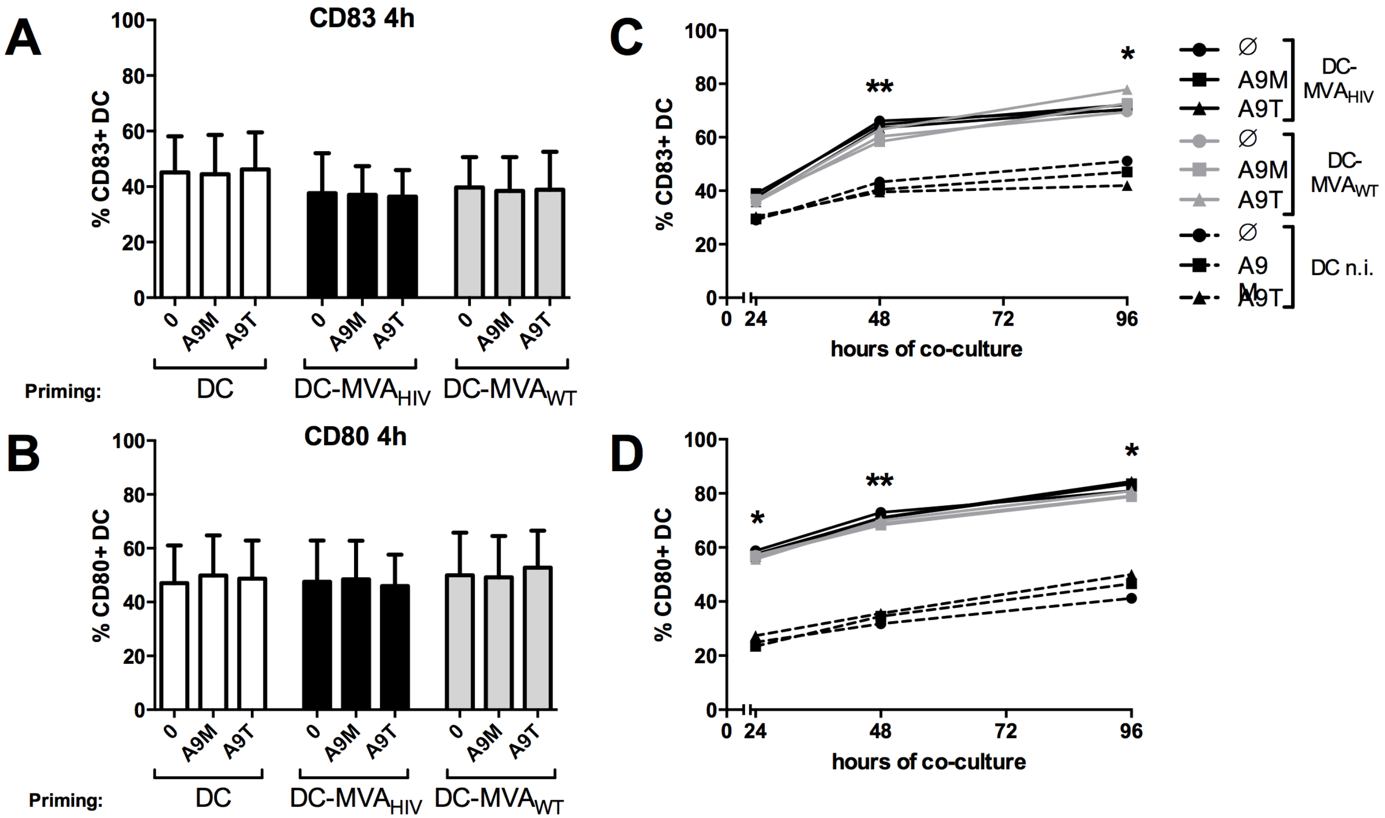

Figure S4 | S100A9-tetramer pre-stimulation does not modify DC maturation.
NK cells were stimulated or not by S100A9 tetramers or S100A9 monomers at 1µg/mL during 4 hours, then NK cells were cultured with DCs infected or not by MVAWT or MVAHIV. The expression of maturation marker CD83 and co-stimulatory molecule CD80 were analyzed on DCs at 4 hours (A and B, n=3), 24h, 48h and 96h of co-culture (C and D, n<4). $^*p \leq 0.05$, $^{**}p < 0.01$ between the expression on non-infected DCs and MVA-infected DCs. A9M: S100A9 monomer; A9T: S100A9 tetramer; DC-MVAWT: MVAWT-infected DC; DC-MVAHIV: MVAHIV-infected DC.
