# Supplementary material for: S100A9 Tetramers, Which are Ligands of CD85j, Increase the Ability of MVAHIV-Primed NK Cells to Control HIV Infection
Source: Front Immunol. 2015 Sep 23;6:478. doi: 10.3389/fimmu.2015.00478 (PMC4585218; doi:10.3389/fimmu.2015.00478)
Supplement: Supplementary file 5 [file Image_5.PDF]

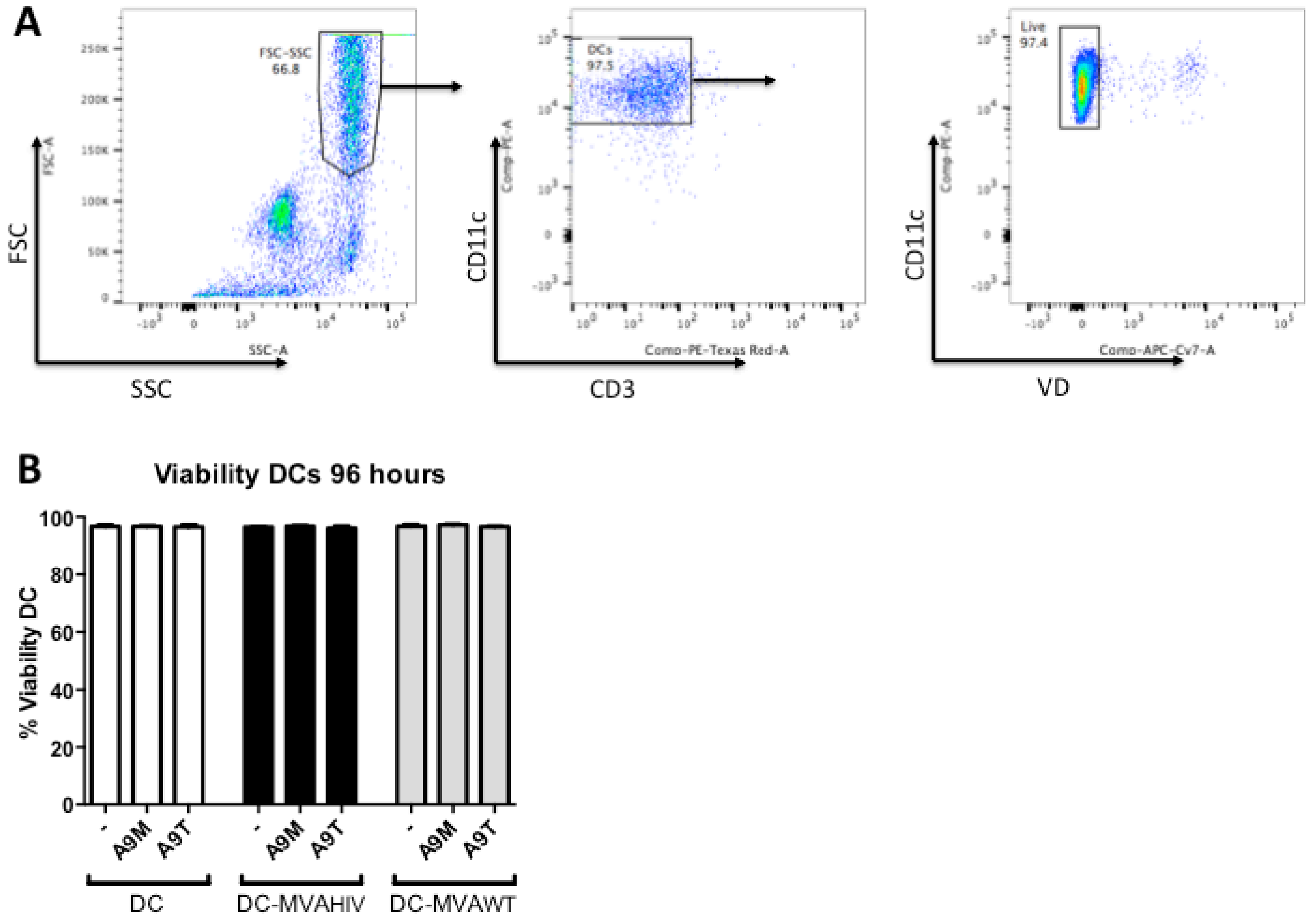

Figure S5 | Viability of DCs in culture with NK cells.
NK cells were stimulated or not by S100A9 tetramers or monomers at 1µg/mL during 4 hours, and cultured with DCs infected or not by MVAWT or MVAHIV, during 4 days. (A) Then, the proportion of live DCs was analyzed by flow cytometry, on gated DCs, using the "Fixable Viability Dye eFluor 780" (eBiosciences). DCs were defined as CD3-CD11c+. (B) Graph shows cumulative results from 3 independent experiments. A9M: S100A9 monomer; A9T: S100A9 tetramer; DC-MVAWT: MVAWT-infected DC; DC-MVAHIV: MVAHIV-infected DC.
