# Supplementary material for: S100A9 Tetramers, Which are Ligands of CD85j, Increase the Ability of MVAHIV-Primed NK Cells to Control HIV Infection
Source: Front Immunol. 2015 Sep 23;6:478. doi: 10.3389/fimmu.2015.00478 (PMC4585218; doi:10.3389/fimmu.2015.00478)
Supplement: Supplementary file 6 [file Image_6.PDF]

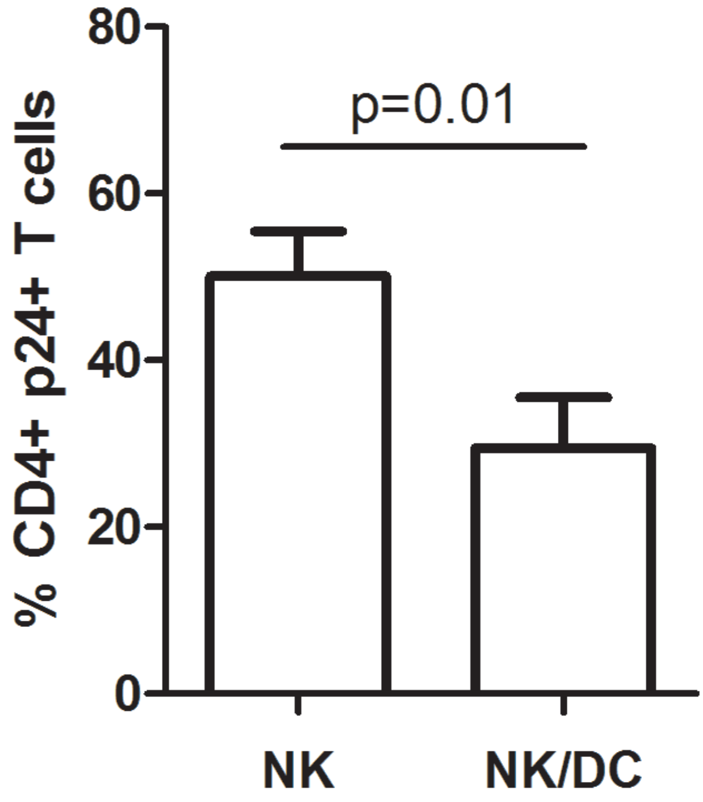

Figure S6 | DCs naturally enhance the ability of NK cells to control HIV infection.
NK cells were cultured or not with DCs during 4 days, at a ratio of 1 NK cell/5 DCs. Then, NK cells were harvested and put in culture with HIV-infected autologous CD4+ T cells. 10 days post-HIV infection we analyzed the percentage of HIV-infected CD4+ T in the culture. Graph shows cumulative results from 9 independent experiments. Results are expressed as mean ± SE, and p value is shown.
