# Supplementary material for: S100A9 Tetramers, Which are Ligands of CD85j, Increase the Ability of MVAHIV-Primed NK Cells to Control HIV Infection
Source: Front Immunol. 2015 Sep 23;6:478. doi: 10.3389/fimmu.2015.00478 (PMC4585218; doi:10.3389/fimmu.2015.00478)
Supplement: Supplementary file 7 [file Image_7.PDF]

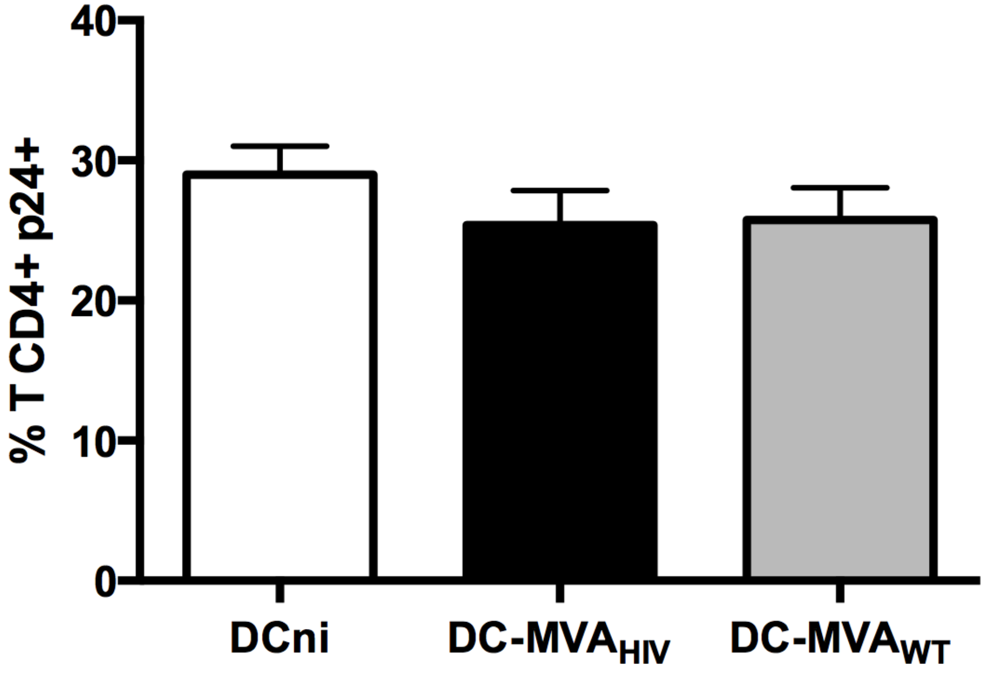


**Figure S7 | DCs exposed to MVAHIV do not decrease HIV infection of CD4+ T cells.**
DCs were infected or not by either MVAHIV or MVAWT. 24 hours later, non-infected DCs were added to a ratio 1:1. After 4 days of co-culture, DCs were harvested and put in culture with HIV-infected autologous CD4+ T cells. 9 days post-HIV infection, the percentage of HIV-infected CD4+ T was analyzed. Graph shows cumulative results from 3 independent experiments. Results are expressed as mean ± SE.
