# Supplementary material for: S100A9 Tetramers, Which are Ligands of CD85j, Increase the Ability of MVAHIV-Primed NK Cells to Control HIV Infection
Source: Front Immunol. 2015 Sep 23;6:478. doi: 10.3389/fimmu.2015.00478 (PMC4585218; doi:10.3389/fimmu.2015.00478)
Supplement: Supplementary file 8 [file Image_8.PDF]

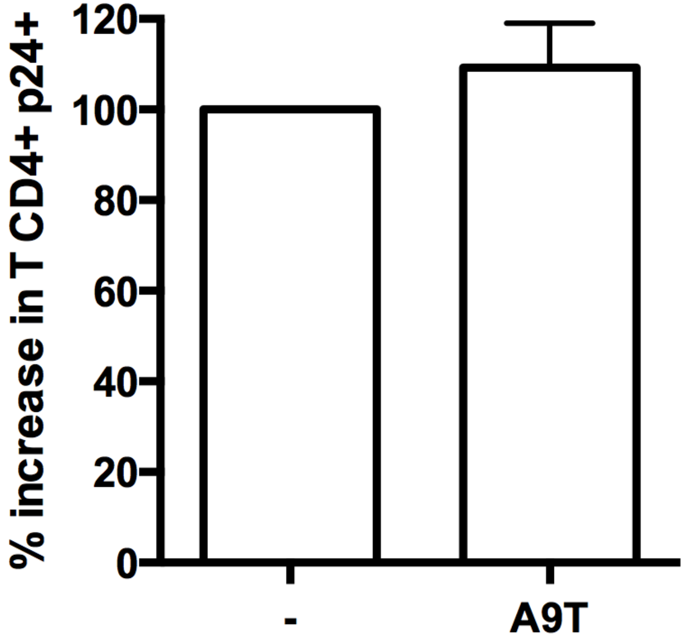

Figure S8 | Stimulation of DCs by S100A9 tetramers does not decrease HIV infection of CD4+ T cells.
DCs were cultured during 4 days in the presence or not of S100A9 tetramers at 1µg/mL, then, DCs were harvested and cultured with HIV-infected CD4+ T cells. Intracellular HIV p24 expression was analyzed on gated CD4+ T cells at day 9 post-HIV infection. Graph shows cumulative percentage increase in HIV p24 expression on CD4+ T cells, from 3 independent experiments. A9T: S100A9 tetramer.
