# Supplementary material for: S100A9 Tetramers, Which are Ligands of CD85j, Increase the Ability of MVAHIV-Primed NK Cells to Control HIV Infection
Source: Front Immunol. 2015 Sep 23;6:478. doi: 10.3389/fimmu.2015.00478 (PMC4585218; doi:10.3389/fimmu.2015.00478)
Supplement: Supplementary file 9 [file Image_9.PDF]

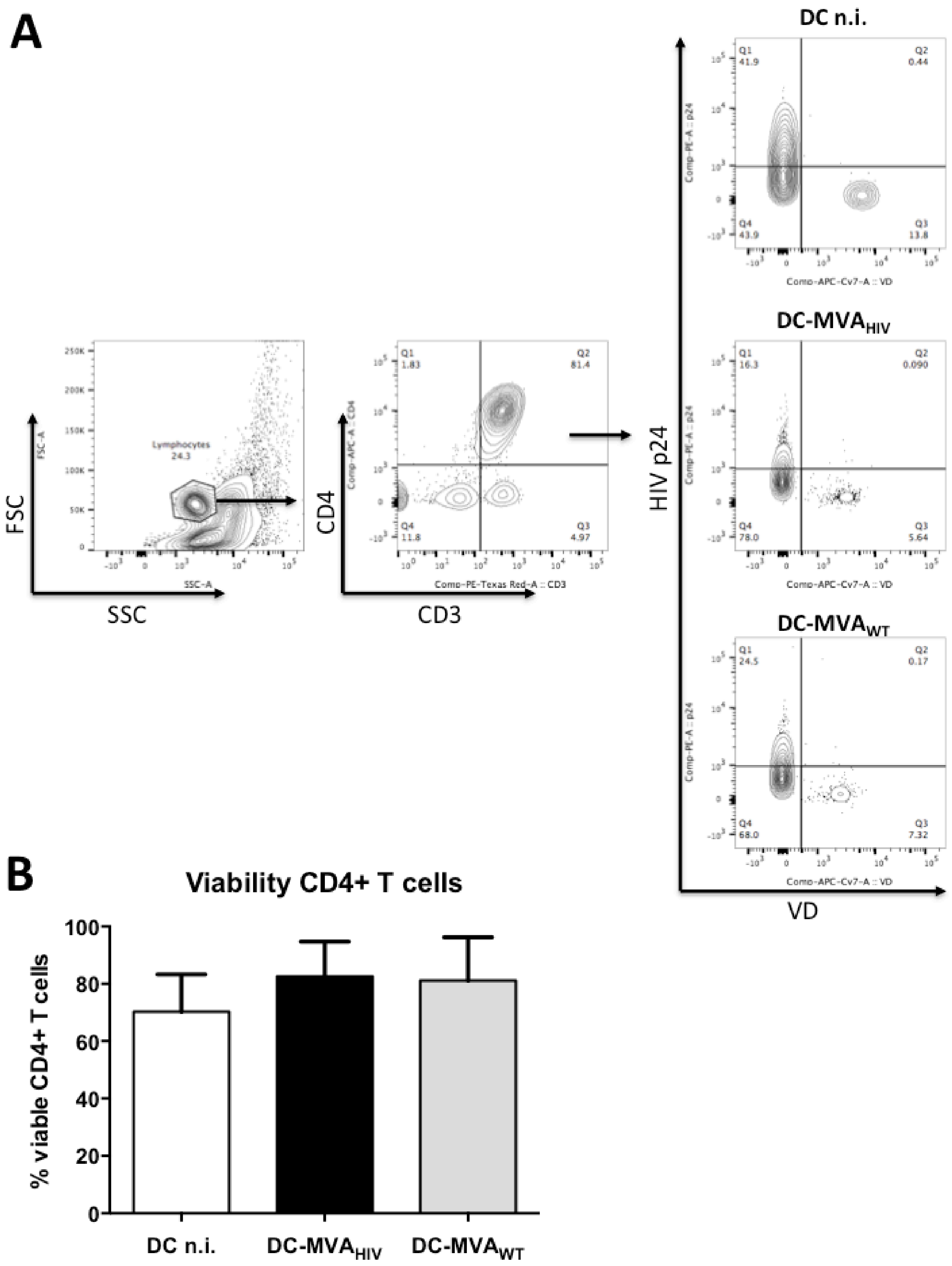

Figure S9 | Viability of CD4+ T cells in culture with primed NK cells.
NK cells were cultured with DCs infected or not by MVAWT or MVAHIV. After 4 days, NK cells were transferred to a culture of HIV-infected CD4+ T cells and the viability of CD4+ T cells at 7 days post-HIV infection was analyzed. (A) Representative analysis of the viability of CD4+ T cells according to the expression of HIV p24. (B) Graph shows cumulative observations of the viability of CD4+ T cells, from 3 independent experiments.
